# Cytoplasmic IGF2BP2 Protein Expression in Human Patients with Oral Squamous Cell Carcinoma: Prognostic and Clinical Implications

**DOI:** 10.7150/ijms.74751

**Published:** 2022-07-04

**Authors:** Shu-Hui Lin, Chiao-Wen Lin, Jeng-Wei Lu, Wei-En Yang, Yueh-Min Lin, Hsueh-Ju Lu, Shun-Fa Yang

**Affiliations:** 1 Department of Surgical Pathology, Changhua Christian Hospital, Changhua, Taiwan; 2 Department of Medical Laboratory Science and Biotechnology, Central Taiwan University of Science and Technology, Taichung, Taiwan; 3 Institute of Oral Sciences, Chung Shan Medical University, Taichung, Taiwan; 4 Department of Dentistry, Chung Shan Medical University Hospital, Taichung, Taiwan; 5 Antimicrobial Resistance Interdisciplinary Research Group, Singapore-MIT-Alliance for Research and Technology, Singapore, Singapore; 6 Institute of Medicine, Chung Shan Medical University, Taichung, Taiwan; 7 Department of Medical Research, Chung Shan Medical University Hospital, Taichung, Taiwan; 8 School of Medicine, Chung Shan Medical University, Taichung, Taiwan; 9 Division of Hematology and Oncology, Department of Internal Medicine, Chung Shan Medical University Hospital, Taichung, Taiwan

**Keywords:** IGF2BP2, tissue microarray, immunohistochemistry, oral squamous cell carcinoma, survival

## Abstract

Oral squamous cell carcinoma (OSCC) is particularly prevalent in Taiwan. The goal of this study was to determine the clinicopathological role of insulin-like growth factor 2 mRNA binding protein 2 (IGF2BP2) proteins as an indicator of clinical outcomes in OSCC patients. In this study, immunohistochemical (IHC) analysis was used to examine IGF2BP2 protein expression in 244 OSCC patients. We investigated the relationships among IGF2BP2 expression, clinicopathological variables, and patient survival. Our results showed that IGF2BP2 cytoplasmic protein expression was significantly correlated with lymph node metastasis, cancer stage, and patient survival. Kaplan-Meier survival curves revealed that elevated cytoplasmic IGF2BP2 expression levels in OSCC patients were associated with poor overall survival. Moreover, multivariate cox proportional hazard models revealed that cytoplasmic IGF2BP2 expression, T status, and lymph node metastasis were independent prognostic factors for survival. In conclusion, IGF2BP2 protein was found to be a helpful predictive marker for OSCC patients, as well as a possible therapeutic target for OSCC treatment.

## Introduction

Oral squamous cell carcinoma (OSCC) is the most common malignant tumor in the head and neck region 1. Tobacco addiction, excessive alcohol consumption, and human papillomavirus (HPV) infection have all been identified as major risk factors for carcinogenesis and the development of OSCC 2. OSCC accounts for more than 90% of all oral cancers, with more than 300,000 new cases and 145,000 fatalities each year 3. Despite advancements in therapy, the 5-year survival rate of OSCC patients remains low 4. Advanced OSCC is characterized by unregulated growth with severe lymphatic metastases and a poor prognosis. As with other forms of cancer, OSCC is caused by a series of complex interactions involving a range of genes and proteins, resulting in a multifactor interaction 5-8. Our lack of understanding as to the molecular processes underlying OSCC development underlines the need to develop new biomarkers as prognostic indicators and treatment targets 9.

Insulin-like growth factor (IGF) and IGF-binding protein play a vital role in the premalignant oral lesions and oral cancer 10-13. Insulin-like growth factor 2 mRNA-binding protein 2 (IGF2BP2) controls IGF2 translation by binding to the 5′ untranslated region (5′UTR) of IGF2 mRNA. In the realm of cancer research, IGF2BP2 is well-known for its regulation of differentiation potential in mouse neocortical neural precursor cells as well as myoblast proliferation, myogenesis, muscle cell motility, and energy consumption 14-16. A number of studies have linked IGF2BP2 gene polymorphisms to the incidence of type 2 diabetes and cancer 17-20. According to one recent study, IGF2BP2 knock-out mice fight obesity by regulating mRNA that encodes for mitochondrial proteins 21.

IGF2BP2 has been shown to promote tumor growth in cases of solid tumors and leukemia 22-27. Recent research has identified IGF2BP2 as a potential oncogene, which, when overexpressed in liver cancer, causes excessive cell proliferation and invasion, resulting in a poor prognosis 28-31. The overexpression of IGF2BP2 has also been shown to promote the development of glioblastoma multiforme by activating the IGF2/phosphoinositide 3-kinase (PI3K)/Akt pathway, thereby making glioblastoma resistant to temozolomide therapy 32. In head and neck squamous cell carcinoma and OSCC tissues, the elevated mRNA or protein expression of IGF2BP2 is indicative of poor prognosis 33-35. The upregulation of IGF2BP2 has also been shown to promote OSCC progression associated with cell proliferation, metastasis, and tumor-infiltrating immune cells 34.

Nonetheless, there is a pressing need to further elucidate the function of IGF2BP2 protein expression and other clinical variables in OSCC. In the current study, immunohistochemical (IHC) analysis was used to examine the expression of IGF2BP2 proteins tissue samples from 244 OSCC patients. We also examined the relationship between IGF2BP2 protein expression and OSCC clinicopathological variables and prognosis. Finally, we sought to identify potential prognostic markers to facilitate the early detection of OSCC.

## Materials and Methods

### Human patients and ethics statement

Patients (*n* = 244) were recruited from Changhua Christian Hospital in Taiwan. The most common forms of treatment included tumor removal and radical neck dissection followed by post-operative irradiation. A number of patients also received 5-fluorouracil (5-FU) and cisplatin chemotherapy. This study was also approved by the Changhua Christian Hospital's Ethics Committee in accordance with Institutional Review Board guidelines (IRB No. 150808, date of approval 03 July 2016). Prior to surgery, all OSCC patients provided written informed consent.

### Tissue microarray preparation and evaluation

In accordance with the methods outlined in previous reports 36, 37, tissue microarrays (TMAs) were created using the OSCC samples, which included typical OSCC tissues and the surrounding epithelial tissue. The samples were fixed with paraffin to perforate tissue cylinders (2 mm in diameter) to produce OSCC and neighboring TMAs using a handmade, semiautomated tissue array. TMAs were created after the pathological evaluation of typical OSCC samples. Two senior pathologists validated the morphology of the malignancy based on representative lesions revealed by staining tissue slices using hematoxylin and eosin (H&E). The American Joint Committee on Cancer (AJCC, 7th Edition) Tumor, Node, Metastasis (TNM) staging system and the Edmondson-Steiner grading system were used to make pathological evaluations of tumor stages and histological differentiation.

### IHC staining and scoring

IHC staining was performed in accordance with our previous studies 38, 39. Following deparaffinization and hydration with various quantities of ethanol, the TMAs were antigen-retrieved using microwave radiation with 0.01 M citrate buffer (pH 6.0) and then incubated in 3 % H_2_O_2_ to block endogenous peroxidase activity, followed by incubation in 10% normal goat serum at 37°C for 1 h. The TMAs were combined with a solution containing monoclonal rabbit anti-human IGF2BP2 antibodies and held at 4°C overnight (Catalog number: ab124930; 1:50 dilution; Abcam, Cambridge, MA, USA). On the following day, the TMAs were tested for immune complex using a LASB 2 Kit (Dako, Carpinteria, CA, USA). The TMAs were stained using aminoethyl carbazole followed by hematoxylin to detect enzyme activity. The experiment involved a positive control (pancreatic cancer tissue as a known positive case) 27 as well as a negative control (samples not treated with the primary antibody) to assess the specificity of IGF2BP2 antibodies for IHC staining.

### Statistical analysis

The clinicopathological variables of cytoplasmic IGF2BP2 protein expression and OSCC were assessed using Fisher's exact test or the Chi-Square test. The Kaplan-Meier method was used to create overall survival curves for OSCC patients with low and high cytoplasmic IGF2BP2 protein expression, and the log-rank test was used to estimate cumulative survival rates. The Cox proportional hazard regression model was used to confirm prognostic variables of OSCC using univariate and multivariate analyses after adjusting the stage, tumor size, lymph node metastasis and cell differentiation status. A p-value of <0.05 was used to identify statistically significant results 36, 37, 40. Statistical Product and Service Solutions (SPSS, version 17) was used for all analysis (SPSS, Inc., Chicago, IL, USA).

## Results

### Demographics and characteristics of human patients with OSCC

Table 1 lists the demographics and data pertaining to patient characteristics. This study included 234 male OSCC patients (95.9%), ranging in age from 32 to 85. Patients were categorized according to disease stage according to criteria outlined by the American Joint Committee on Cancer (AJCC), as follows: stage I (n=43; 17.6%), stage II (n=54; 22.1%); stage III (n=29; 11.9%), and stage IV (n=118; 48.4%). The tumor size distribution was as follows: tumor size I (T1) (n=57; 23.4 %), tumor size II (T2) (n=78; 31.9 %), tumor size III (T3) (n=19; 7.80 %), and tumor size IV (T4) (n = 90; 36.9%). Patients were also categorized according to histological grade, as follows: well differentiated (Well; n=39; 16.0%), moderately differentiated (Moderate; n=198; 81.1%), and poorly differentiated (Poor; n=7; 2.9%).

### IGF2BP2 protein expression in OSCC and clinicopathological variables

**Figure 1:**
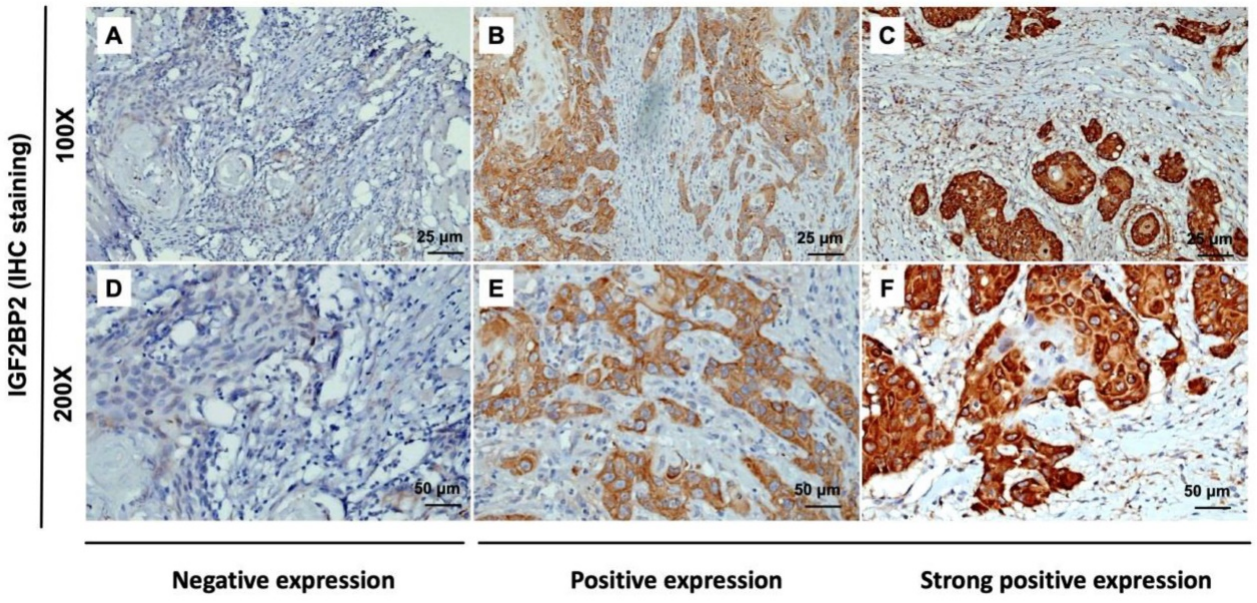
IHC analysis of cytoplasmic IGF2BP2 expression in human OSCC tissue showing negative (A and D), positive (B and E), and strong positive expression (C and F). Magnification: (top panel) 100x and lower panel (200x). Scale bars=25 and 50 µm.

The expression of IGF2BP2 in OSCC cancer tissue was examined via IHC staining. As shown in Figure 1, samples were divided into two groups based on IGF2BP2 protein expression, as follows: (1) Low cytoplasmic staining of IGF2BP (negative expression); (2) high cytoplasmic staining of IGF2BP (positive and strong positive expression). The tissue samples were stratified as follows: Low IGF2BP2 expression (n=209; 85.7%) and high IGF2BP2 expression (n=35; 14.3%). The relationship between IGF2BP2 expression and clinicopathological variables in individuals with OSCC was used as the clinical basis in assessing the clinical relevance of IGF2BP2 protein expression using the Fisher exact test or the Chi-square test (Table 2). High IGF2BP2 expression was significantly linked to lymph node metastases, disease stage, and the survival of human patients with OSCC (p=0.004, p=0.027, p=0.038, and p=0.011, respectively). In OSCC patients, we did not observe a significant relationship between IGF2BP2 expression and age, histological grade, T status, distant metastasis, smoking, or betel quid chewing.

### High IGF2BP2 protein expression levels are linked to shorter overall survival in OSCC patients

**Figure 2:**
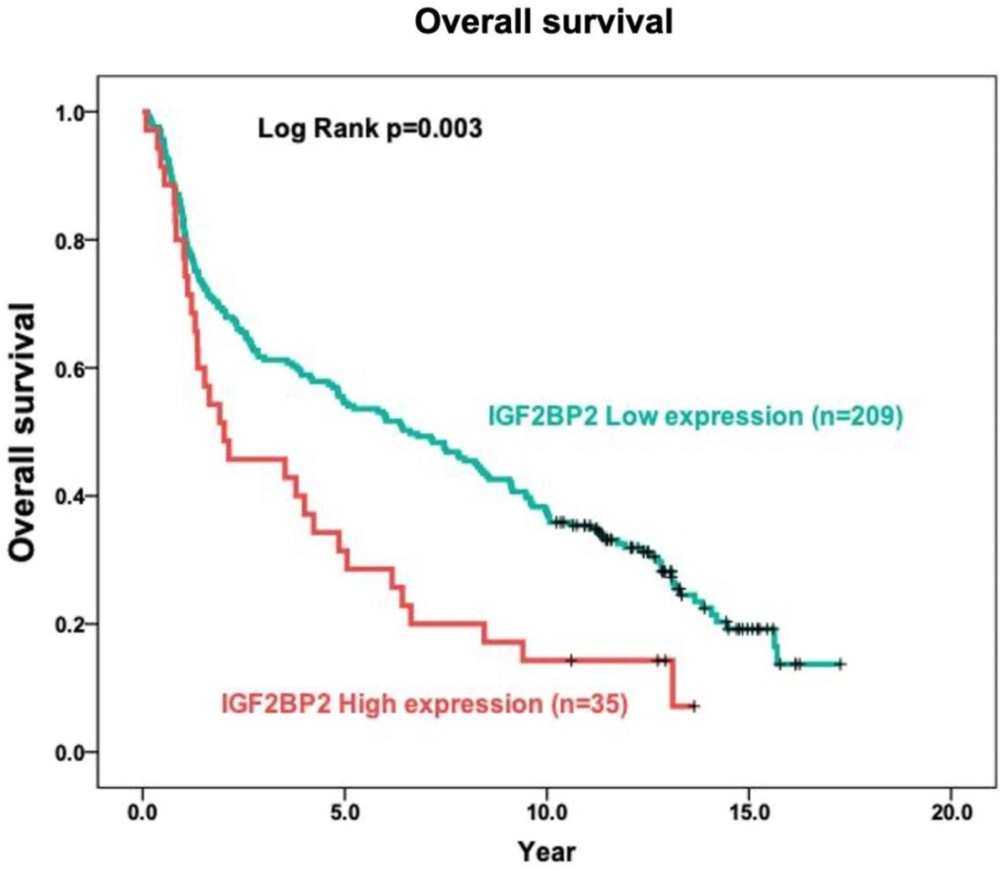
Relationship between cytoplasmic IGF2BP2 expression levels and overall survival in patients with OSCC based on the Kaplan-Meier method. Analysis was based on included 244 oral squamous cell carcinoma samples, using Kaplan-Meier analysis in conjunction with the log-rank test to establish survival curves.

The role of IGF2BP2 in tumor prognosis was elucidated in terms of the relationship between IGF2BP2 expression and the overall survival of OSCC patients. In Kaplan-Meier analysis, the survival curves of OSCC patients with high IGF2BP2 expression were lower than those with low IGF2BP2 expression (p=0.003) using log-rank tests (Figure 2).

### Prognostic indicators of clinicopathological variables and IGF2BP2 protein expression in OSCC patients identified using Cox proportional-hazards models

Univariate and multivariate analysis based on the Cox proportional-hazards model were used to determine the degree to which independent prognostic factors of IGF2BP2 expression affect overall survival of OSCC patients (Table 3). Univariate and multivariate analyses both revealed that the overall survival rate of OSCC patients was significantly linked to the expression of IGF2BP2 (p=0.003, 95% CI 1.213 to 2.644; p=0.039, 95% CI 1.530 to 2.289, respectively), histological grade (p=0.039, 95% CI 1.021 to 2.218), T status (p<0.001, 95% CI 1.239 to 2.139; p=0.013, 95% CI 1.132 to 2.887, respectively), lymph node metastasis (p<0.001, 95% CI 1.384 to 2.423; p=0.012, 95% CI 1.181 to 2.435, respectively) and stage (p<0.001, 95% CI 1.342 to 2.373).

## Discussion

Cancer has become one of the most common causes of mortality among middle-aged and elderly individuals. OSCC is among the most common malignant tumors of the head and neck. The high recurrence and metastasis of the disease pose a severe threat to human health and welfare 41-43. OSCC is generally detected in the middle or late stages, due to non-specific early clinical symptoms. Despite recent advancements in the treatment of OSCC, the 5-year survival rate remains low 44, 45. OSCC is a malignant tumor affecting the head and neck region, which has also been shown to harm oral epithelial cells 46. OSCC has been linked to genetic modifications, including mutations to chromosomes 3, 9, 11, and 13 47, 48. Increasing survival rates and improving the quality of life of OSCC patients will depend on the identification of molecular biomarkers and therapeutic techniques for the diagnosis and treatment of OSCC 49.

Note that the specific mechanism underlying OSCC tumorigenesis has yet to be elucidated, and there are currently no reliable early indicators for the diagnosis or prognosis of OSCC 8, 50. Identifying genes with distinct patterns of expression in OSCC tumors versus normal tissue could advance our understanding of OSCC etiology, while providing important diagnostic markers and therapeutic targets for OSCC therapy 51. The aberrant expression of oncogenes and tumor suppressor genes has previously been demonstrated to have anti-tumor or tumor-promoting effects 52. Several biomarkers have been linked to OSCC occurrence and disease progression, indicating that they play an important role in carcinogenesis. In the current study, we assessed IGF2BP2 protein expression levels within the context of the prognosis of OSCC patients.

Researchers have reported that IGF2BP2 is elevated in cases of malignancy. IGF2BP2 levels can also use as a prognostic indicator of acute myelocytic leukemia 25, breast cancer 53, endometrial adenocarcinoma 54, liposarcoma 55, pancreatic cancer 27, hepatocellular carcinoma 30 and OSCC 35. Moreover, Lu et al. reported that IGF2BP2 may play an important role in the development of ESCC carcinogenesis 56. In the current study, we discovered the overexpression of IGF2BP2 in OSCC patients (Figure 1), which is consistent with previous findings 35. We also determined that IGF2BP2 overexpression is related to poor overall survival outcomes in OSCC patients (Figure 2), which suggests that it could perhaps be used as a prognostic indicator for use in OSCC risk classification. Elevated IGF2BP2 expression in OSCC cells has been linked to cell proliferation, metastasis, and tumor-infiltrating immune cells in *in vitro* experiments 34. Those studies confirm our clinical results, which suggest that IGF2BP2 enhances OSCC epithelial cell proliferation and epithelial-mesenchymal transition (EMT), thereby promoting tumor growth and invasion in OSCC patients.

In the current study, we used clinical tissue samples from OSCC patients to characterize the connection between IGF2BP2 and clinicopathologic indicators. High cytoplasmic IGF2BP2 expression was strongly linked to disease stage and survival. The connections between positive IGF2BP2 protein expression and lymph node metastases, as well as between IGF2BP2 and AJCC cancer stage, suggest that IGF2BP2 may play a role in OSCC metastasis (Table 2). Our findings are consistent with previous studies in which IGF2BP2 mRNA expression levels were examined in the context of clinicopathological characteristics based on public clinical datasets 35. Univariate and multivariate analyses both identified IGF2BP2 expression, histological grade, T status, lymph node metastases, and disease stage as key independent prognostic factors impacting the overall survival of OSCC patients (Table 3). Our findings suggest that IGF2BP2 may operate as an oncogene in OSCC cells, which is in line with earlier research 34, 35. Note that this was the first study to examine the use of clinicopathological variables and IGF2BP2 protein expression within the context of OSCC prognosis. Further multistep research, including both *in vitro* and *in vivo* testing, will be required to corroborate our data and assess the efficacy of IGF2BP2 as a therapeutic target.

In conclusion, our findings demonstrate that IGF2BP2 protein expression is prevalent in OSCC tissues, and that protein expression levels were associated with histological grade, T status, lymph node metastasis, disease stage, and survival. Our results from 244 OSCC patients show a strong link between IGF2BP2 protein levels and survival rates. Our results also show that IGF2BP2 protein expression could potentially be used as an independent OSCC prognostic predictor and/or therapeutic target for OSCC treatment.

## Figures and Tables

**Table 1:** Demographics and characteristics of human patients with oral squamous cell carcinoma

Factors	(n = 244)	Percentage
Gender		
Male	234	95.9%
Female	10	4.10%
Age (yrs)		
Range	32-85	
Mean	55.0	
Medium	53.0	
AJCC cancer stage		
I	43	17.6%
II	54	22.1%
III	29	11.9%
IV	118	48.4%
T (Tumor size)		
T1	57	23.4%
T2	78	31.9%
T3	19	7.8%
T4	90	36.9%
N (Lymph node)		
No	151	61.9%
Yes	93	38.1%
M (Metastasis)		
No	242	99.2%
Yes	2	0.80%
Histological grade (differentiation)		
Well	39	16.0%
Moderate	198	81.1%
poor	7	2.9%

**Table 2:** Clinicopathologic variables correlated with IGF2BP2 expression in human patients with oral squamous cell carcinoma

Variables	Cytoplasmic Staining of IGF2BP2			
	Low	High	(n=244)	p-value^a^
Age (yrs)	55.1±10.9	54.3±10.0		0.331
Gender				
Male	200 (95.7%)	34 (97.1%)	234	1.000^a^
Female	9 (4.3%)	1 (2.9%)	10	
Smoking				
No	69 (39.7%)	8 (30.8%)	77	0.385
Yes	105 (60.3%)	18 (69.2%)	123	
Betel quid chewing				
No	56 (40.9%)	7 (36.8%)	63	0.737
Yes	81 (59.1%)	12 (63.2%)	93	
AJCC cancer stage				
I, II	89 (42.6%)	8 (22.9%)	97	0.027*
III, IV	120 (57.4%)	27 (77.1%)	147	
T (Tumor size)				
T1/T2	117 (56.0%)	18 (51.4%)	135	0.616
T3/T4	92 (44.0%)	17 (48.6%)	109	
Lymph node metastasis				
No	137 (65.6%)	14 (40.0%)	151	0.004*
Yes	72 (34.4%)	21 (60.0%)	93	
Distant metastasis				
No	207 (99.0%)	35 (100%)	242	1.000^a^
Yes	2 (1.0%)	0 (0%)	2	
Histological grade (differentiation)				
Well	37 (17.7%)	2 (5.7%)	39	0.083^ a^
Moderate/Poor	172 (82.3%)	33 (94.3%)	205	
Survival				
≤4 year	86 (41.1%)	21 (60.0%)	107	0.038*
>4 year	123 (58.9%)	14 (40.0%)	137	
≤5 year	95 (45.5%)	24 (68.6%)	119	0.011*
>5 year	114 (54.5%)	11 (31.4%)	125	

**^a^**The p-value using Fisher's exact test or Chi-square test. *p<0.05

**Table 3:** Overall survival and clinicopathologic variables of human patients with oral squamous cell carcinoma using univariate and multivariate analysis

Variables (n = 244)	Univariate analysis		Multivariate analysis	
	Hazard ratio (95% CI)^a^	p-value	Hazard ratio (95% CI)^a^	p-value
Expression of IGF2BP2				
Low	1.0	0.003*	1.0	0.039*
High	1.79 (1.213-2.644)		1.53 (1.530-2.289)	
AJCC cancer stage				
I, II	1.0	<0.001*	1.0	0.642
III, IV	1.78 (1.342-2.373)		0.88 (0.498-1.537)	
T (Tumor size)				
T1/T2	1.0	<0.001*	1.0	0.013*
T3/T4	1.63 (1.239-2.139)		1.81 (1.132-2.887)	
Lymph node metastasis				
No	1.0	<0.001*	1.0	0.012*
Yes	1.83 (1.384-2.423)		1.65 (1.181-2.435)	
Histological grade (differentiation)				
Well	1.0	0.039*	1.0	0.136
Moderate/Poor	1.51 (1.021-2.218)		1.38 (0.904-2.103)	

95% CI: 95% Confidence interval; ^a^Hazard ratio was adjusted for gender and age. *p<0.05
